# The Automatic Neuroscientist: A framework for optimizing experimental design with closed-loop real-time fMRI

**DOI:** 10.1016/j.neuroimage.2016.01.032

**Published:** 2016-04-01

**Authors:** Romy Lorenz, Ricardo Pio Monti, Inês R. Violante, Christoforos Anagnostopoulos, Aldo A. Faisal, Giovanni Montana, Robert Leech

**Affiliations:** a The Computational, Cognitive and Clinical Neuroimaging Laboratory, Division of Brain Sciences, Imperial College London, London W12 0NN, UK; b Department of Bioengineering, Imperial College London, London SW7 2AZ, UK; c Department of Mathematics, Imperial College London, London SW7 2AZ, UK; d Department of Biomedical Engineering, King's College London, London SE1 7EH, UK

**Keywords:** Experimental design, Real-time fMRI, Machine learning, Bayesian optimization, Closed-loop, Brain–computer interface

## Abstract

Functional neuroimaging typically explores how a particular task activates a set of brain regions. Importantly though, the same neural system can be activated by inherently different tasks. To date, there is no approach available that systematically explores whether and how distinct tasks probe the same neural system. Here, we propose and validate an alternative framework, the *Automatic Neuroscientist*, which turns the standard fMRI approach on its head. We use real-time fMRI in combination with modern machine-learning techniques to automatically design the optimal experiment to evoke a desired target brain state. In this work, we present two proof-of-principle studies involving perceptual stimuli. In both studies optimization algorithms of varying complexity were employed; the first involved a stochastic approximation method while the second incorporated a more sophisticated Bayesian optimization technique. In the first study, we achieved convergence for the hypothesized optimum in 11 out of 14 runs in less than 10 min. Results of the second study showed how our closed-loop framework accurately and with high efficiency estimated the underlying relationship between stimuli and neural responses for each subject in one to two runs: with each run lasting 6.3 min. Moreover, we demonstrate that using only the first run produced a reliable solution at a group-level. Supporting simulation analyses provided evidence on the robustness of the Bayesian optimization approach for scenarios with low contrast-to-noise ratio. This framework is generalizable to numerous applications, ranging from optimizing stimuli in neuroimaging pilot studies to tailoring clinical rehabilitation therapy to patients and can be used with multiple imaging modalities in humans and animals.

## Highlights

• Mapping between cognitive task and neural processes is often not straightforward.
• Current fMRI approach is not sufficient for holistic investigation of this mapping.
• We propose a novel framework combining real-time fMRI and optimization techniques.
• We validate our closed-loop method and find it to be highly efficient and accurate.
• This framework is generalizable to numerous innovative applications.

## Introduction

Understanding how cognition and the brain interrelate is a central aim of functional neuroimaging. The standard approach only takes a limited view by probing the neural system associated with one or a few task conditions; i.e., a neuroscientist designs a task/stimulus and investigates the brain regions that respond to it. However, this approach does not answer whether and how the same neural system can be activated by many, often quite different tasks. In the literature, the same network of brain regions has frequently been ascribed with completely diverse functional descriptions based on the different tasks that evoked it, e.g., ‘the pain matrix’ (Davis, 2000, Treede et al., 1999, Wager et al., 2013) is very similar to the ‘salience network’ (evoked by cognitively surprising stimuli) (Seeley et al., 2007, Uddin, 2015). Similarly, the superior temporal sulcus has been termed the ‘chameleon of the brain’ (Hein and Knight, 2008) due to its involvement in different functional roles, ranging from audio–visual integration (Amedi et al., 2005) to motion (Puce and Perrett, 2003), speech (Price, 2000) and face processing (Haxby et al., 1999). Therefore, from a theoretical perspective, only considering how one or a few tasks activate a given region makes understanding its functional role as difficult as finding a needle in a haystack; we do not know which task activates a specific brain region and how it compares to all the other possible tasks. It can also give rise to the reverse inference problem (Poldrack, 2006), where cognitive functions are ascribed to a region because that region has been activated for a task previously. Gaining insight into the complex relationship between the brain and cognition therefore requires a more holistic investigation of the many tasks-to-many brain regions mapping.

To date, this question can only be partially addressed by using post-hoc meta-analyses synthesizing many studies involving different tasks (Poldrack, 2006, Yarkoni et al., 2011, Yeo et al., 2014). Limitations of meta-analyses range from differences in hardware, pre-processing and analysis pipelines, experimental design, subject sample composition (Costafreda, 2009) to the coarse cognitive ontology in the databases (Poldrack, 2006). In addition, powerful automated meta-analytic frameworks cannot extract information about fine-grained cognitive states, thus are primarily useful for large-scale analyses involving broad cognitive domains (Yarkoni et al., 2011). Importantly, meta-analyses are based on group level results, where inter-subject variability is considered as an effect to overcome. There is no approach available that systematically explores whether and how distinct tasks probe the same neural system within an individual. Individual differences in regional activation for a given task may reflect a different involvement for that region in different tasks and hence may open new avenues for investigating individual differences in neural function or in understanding neurological or psychiatric disorders.

**Fig. 1:**
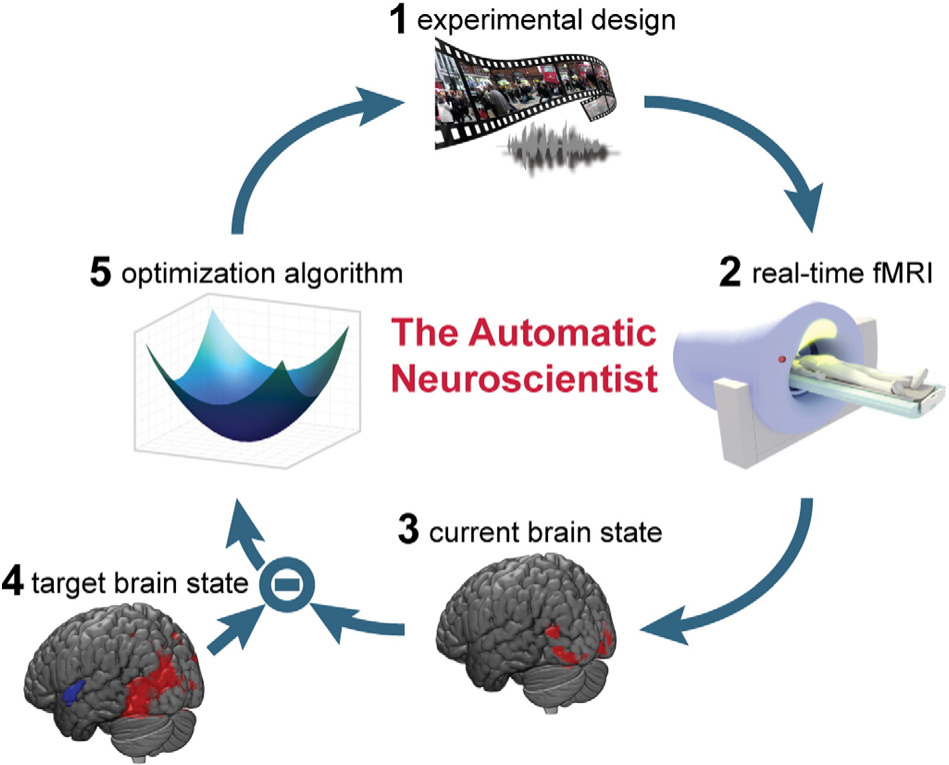
High-level overview of the *Automatic Neuroscientist*. The optimization algorithm starts with selecting a random parameter vector from the available experiment parameter space. (1) The parameter vector determines an experimental condition (e.g. an audio–visual stimulus combination) that is presented to the subject. (2) Whole-brain functional images are acquired and analyzed in real-time in response to the stimulus. (3) Information about the current brain state is extracted and (4) compared to the pre-defined target brain state. This result is then fed into the optimization algorithm. (5) Based on this, the optimization algorithm chooses a parameter vector closer to the minimum of the objective function, hence trying to optimize for the target brain state. This closed-loop cycle then continues until some stopping criterion is reached.

Here, we propose and validate an innovative framework that turns the typical functional magnetic resonance imaging (fMRI) approach on its head to better address the many tasks-to-region mapping: The *Automatic Neuroscientist*. This framework uses real-time fMRI in combination with state-of-the-art optimization techniques to automatically adjust the experimental conditions. The *Automatic Neuroscientist* starts with a target brain state and finds a set of task/stimuli that maximally activates it. This is done in a closed-loop fashion (Fig. 1), i.e. the subject's brain state in response to the current experimental condition is analyzed in real-time and evaluated against the pre-defined target brain state. Based on this, the algorithm proposes the stimulus that will be presented to the subject in the next iteration. This cycle continues until the optimal experimental condition is found.

**Fig. 2:**
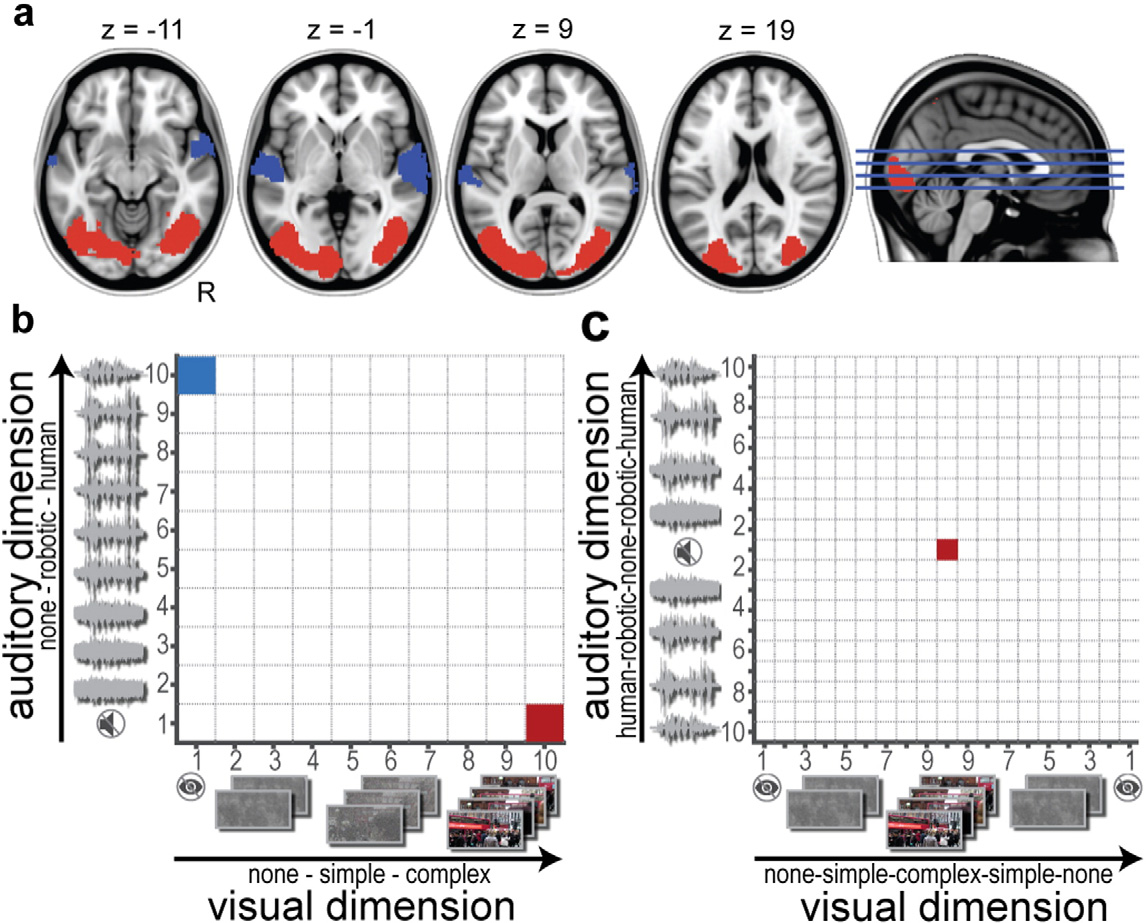
Target brain state and two-dimensional experiment parameter space for both studies. (a) Based on a previous study (Braga et al., 2013) we identified two target brain regions: bilateral lateral occipital cortex (red) and bilateral superior temporal cortex (blue) that strongly activate for complex visual (e.g., naturalistic movie) or auditory stimuli (e.g., speech), respectively. The two tested target brain states of interest were: (1) maximized occipital cortex activity with minimum superior temporal cortex activity and (2) maximized superior temporal cortex with minimized occipital cortex activity. (b) Parameter space of Study 1 with 10 × 10 (100) possible combinations composed of auditory and visual stimuli of varying complexity. The optimization algorithm traversed through the two-dimensional parameter space in order the find the most optimal audio–visual stimulus combination. Based on previous work (Braga et al., 2013), the hypothesized optimal stimulus combination for evoking target brain state (1) is the most complex visual stimulus in combination with no auditory input (red square). The reverse stimulus combination (complex auditory, no visual input) was hypothesized to be optimal for target brain state (2) (blue square). (c) The larger and more challenging parameter space of Study 2 involving 19 × 19 (361) possible combinations. Here, stimuli were only optimized for target brain state (1). The hypothesized optimal stimulus combination is now, due to mirroring of the axes, located in the center of the grid (red square).

We have conducted two proof-of-principle studies involving visual and auditory stimuli as well as optimization algorithms of varying complexity. In both studies, the target brain state was simply defined as the difference in brain activity between two pre-specified target brain regions: the bilateral lateral occipital cortex and bilateral superior temporal cortex (Fig. 2a). Importantly though, this framework can be extended to any desirable target brain state. For example, the stimuli could also be optimized to maximize a specific functional connectivity network configuration measured ‘on-the-fly’ (Monti et al., 2015, Monti et al., 2014). Our framework was validated in both studies by using well-known optimal visual and auditory stimuli that have been shown to strongly activate the target brain regions (Braga et al., 2013). We modified these stimuli to span an extensive experiment parameter space consisting of visual and auditory stimuli that varied in their complexity (Fig. 2b–c). Based on previous work (Braga et al., 2013), we strongly hypothesized a certain audio–visual stimulus combination to optimally evoke the target brain state.

The experiment parameter space is defined a priori, before conducting the actual experiment. Therefore, the *automated* component of our proposed approach is the process of automatically traversing the extensive experiment parameter space to learn the combination of audio–visual stimuli that best evokes the target brain state in a fully closed-loop and self-regulated manner. There are two fundamental challenges posed for such a task. First, the objective function is not available analytically nor can we make formal statements regarding its properties (for example convexity). This therefore precludes the use of traditional optimization methods and we are forced to employ methods that rely only on measurements of the objective function (i.e., empirical data). Second, the presence of non-neural noise (with both physiological and non-physiological origins) is well documented for fMRI experiments; therefore noise robust methods are vital.

In order to address both of these issues, we used a stochastic approximation approach (i.e., SPSA) in Study 1 and a non-parametric Bayesian optimization approach in Study 2. The motivation behind applying two different optimization algorithms is based on the distinct aims of the two studies. Study 1 was designed to simply demonstrate that our approach could accurately find the audio–visual stimulus combination that optimizes brain activity in relation to a target brain pattern. For this case, stochastic approximation algorithms have the clear strength of making very limited assumptions regarding the data. However, the aim of the subsequent Study 2 was to go beyond simple convergence onto the optimal experiment conditions and to demonstrate how our framework can be used to rapidly map out the underlying relationship between stimuli and neural responses across an extensive experiment parameter space. For this case, stochastic approximation approaches are ill suited as they relegate the objective to only learning the optimal stimulus pairing as opposed to obtaining a far richer understanding of the global relationships between stimuli and neural response across experiment conditions. Moreover, stochastic approximation methods estimate the gradient at every iteration without exploiting information about estimates from any previous observation, which increases the susceptibility to noisy outliers and reduces the efficiency in low contrast-to-noise scenarios. Hence, in Study 2 we used a non-parametric Bayesian optimization approach. Supporting simulation analyses were carried out to demonstrate the robustness of the Bayesian optimization for a range of contrast-to-noise ratios.

We show that our closed-loop framework is far more efficient than the standard approach in that it substitutes an otherwise exhaustive search through all possible tasks by performing an optimal search across many experimental dimensions simultaneously before converging on the optimal experimental setup to evoke a desired pattern of brain activation. Moreover, this framework provides a description of the whole experiment space under investigation, meaning the complex relationship between task and brain can be unveiled more easily.

## Methods

### Subjects

Twelve healthy volunteers (7 females, mean age ± SD: 26.8 ± 4.5 years) participated in our studies. Subjects had no history of either contraindication to MRI scanning or neurological/psychiatric disorders. Subjects had normal or corrected-to-normal vision and gave written informed consent for their participation. The study was approved by the Hammersmith Hospital (London, UK) research ethics committee. Subjects were informed about the real-time nature of the fMRI scans but no information was given on the actual aim of the study or which parameters in the experiment would be adapted in real-time. Most importantly, subjects were unaware of the target brain state our algorithm was optimizing for.

### Target brain state and scanning conditions

We identified two target brain regions: bilateral lateral occipital cortex and bilateral superior temporal cortex (Fig. 2a). Masks for these two brain regions were obtained from thresholded (z > 5) and binarized group-level maps (Braga et al., 2013). For Study 1, seven subjects (5 females, mean age ± SD: 26.7 ± 5.3 years) underwent two separate real-time fMRI runs. The runs only differed in the a-priori defined target brain state. The two tested target brain states of interest were: (1) maximized occipital cortex activity with minimum superior temporal cortex activity; and (2) maximized superior temporal cortex with minimized occipital cortex activity. The order of runs was counterbalanced across participants. For Study 2, five new subjects were recruited (2 females, mean age ± SD 26.8 ± 3.6 years). Four of them underwent four separate real-time fMRI runs while we were only able to record a single run in one subject due to technical failure of the scanner. As Study 1 demonstrated that our framework works for either target brain state (i.e. target brain state (1) and (2), see Results section), we simplified the design of Study 2 by only optimizing for target brain state (1) in all runs of Study 2.

### Experiment parameter space

The range of all potential experimental conditions was mapped onto a two-dimensional parameter space with each dimension corresponding to audio or visual stimuli, respectively (see Fig. 2b/c for the experiment parameter space used in Study 1/Study 2). For Study 1, we defined a parameter space with 100 possible audio–visual stimuli combinations. For both modalities, stimuli varied in complexity from no visual input (black background) to a moving naturalistic street scene or from no audio input to a spoken sentence by a human voice in 10 discrete steps, respectively (Fig. 2b). Based on prior literature (Braga et al., 2013), we strongly hypothesized that our target brain state (1) would be evoked by the most complex visual stimulus in combination with no auditory input (see red square in the bottom right corner in Fig. 2b) while the reverse (complex auditory, no visual) was expected for our target brain state (2) (see blue square in the top left corner in Fig. 2b). For Study 2, we used a similar two-dimensional experiment parameter space; however, this time we expanded the parameter space described in Study 1 by mirroring the visual and auditory axes. This was motivated by the aim to demonstrate the performance of the *Automatic Neuroscientist* using an even more challenging experiment parameter space with 361 possible states: 19 discrete steps along the visual dimensions and 19 discrete steps along the auditory dimension (Fig. 2c). The hypothesized optimal stimulus combination (i.e., most complex visual stimulus in combination with no auditory input) for evoking target brain state (1) was now located in the center of the grid at coordinate [10 10] (see red square in Fig. 2c).

#### Visual stimuli

For visual stimuli we used color video footage displaying a naturalistic street scene previously used in Braga et al. (2013). Stimuli varying parametrically were generated by altering the following features of the video: number of frames (in which a lower number of frames is subjectively experienced as a slower video), video image size, image saturation, spatial blurring (using a 2D Gaussian filter of constant size with varying SD) and varying amounts of added Gaussian white noise (with zero mean and varied variance) (see Supplementary Table 1). For examples of the parametrically varied visual stimuli used in the study, please refer to Supplementary Fig. 1.

#### Auditory stimuli

For auditory stimuli, we used four sentences spoken by one male speaker retrieved from the Australian National Database of spoken language (Millar et al., 1994). Parametrically varying stimuli were generated using noise-vocoded speech, created with Praat (Boersma, 2001). Noise-vocoded speech was created by dividing the spoken sentence into varying numbers of logarithmically-spaced frequency bands. For each frequency band the amplitude envelope was extracted, and then used to modulate noise in the respective frequency band. Finally, the frequency bands are recombined to create the noise-vocoded sentence. To increase parametric variation amongst the stimuli, we added additional Gaussian noise (with zero mean and varied SD) to the original sentence before noise-vocoding was performed (see Supplementary Table 2). To reduce adaptation effects of the stimuli, we randomly selected two out of four sentences for each run.

Each audio–visual stimulus presented to the subjects was chosen from this extensive experiment parameter space by the optimization algorithm. In both studies, subjects were presented with audio–visual stimuli in blocks of 10 s followed by 10 s of no stimulus baseline (black background). Stimuli were presented in the center of the screen with a black background using the MATLAB Psychophysics Toolbox (Psychtoolbox-3 (Brainard, 1997, Pelli, 1997)). Subjects were instructed to actively attend to any auditory or visual stimuli without prioritizing one modality over the other.

### Real-time fMRI

Whole-brain coverage images were acquired in real-time by a Siemens Verio 3 T scanner using an EPI sequence (T2*-weighted gradient echo, voxel size: 3.00 × 3.00 × 3.00 mm, field of view: 192 × 192 × 105 mm, flip angle: 80°, repetition time (TR)/echo time (TE): 2000/30 ms, 35 interleaved slices with 3.00 mm thickness). Prior to the online run, a high-resolution gradient-echo T1-weighted structural anatomical volume (reference anatomical image (RAI), voxel size: 1.00 × 1.00 × 1.00 mm, flip angle: 9°, TR/TE: 2300/2.98 ms, 160 ascending slices, inversion time: 900 ms) and one EPI volume (reference functional image (RFI)) were acquired. Offline and online pre-processing were carried out with FSL (Jenkinson et al., 2012). The first steps occurred offline prior to the real-time fMRI scan. Those comprised brain extraction of the RAI and RFI using BET (Smith, 2002) followed by an affine co-registration of the RFI to the RAI and subsequent linear registration (12 DOF) to a standard brain atlas (MNI) using FLIRT (Jenkinson et al., 2002, Jenkinson and Smith, 2001). The resulting transformation matrix was used to register the two target brain masks from MNI to the functional space of the respective subject. For online runs, incoming EPI images were motion corrected in real-time using MCFLIRT (Jenkinson et al., 2002) with the previously obtained RFI acting as reference. In addition, images were spatially smoothed using a 5 mm FWHM Gaussian kernel. Means of the target brain regions for each TR were simultaneously extracted using a general linear model (GLM) approach. The second stage of pre-processing involved cleaning the two extracted timecourses in real-time by removing low-frequency signal drifts with an exponential moving average (EMA with smoothing factor α = 0.96, time constant τ = 49 s, high-pass filter cut-off frequency = 0.003 Hz). In addition, high-frequency noise and large signal spikes were removed with a modified Kalman filter. Both algorithms were obtained from Koush et al. (2012). The pre-processed timecourses were written into a separate text file for subsequent analyses. The experiment commenced after 10 TRs to allow for T1 equilibration effects.

### SPSA optimization

For Study 1, we used the Simultaneous Perturbation Stochastic Approximation (SPSA) algorithm (Spall, 1998). A series of modifications were made in order to address the discrete nature of the experiment parameter space (Gerencsér et al., 1999). SPSA algorithms are stochastic approximations that do not require analytic information about the nature of the objective function. At each iteration, the algorithm randomly proposes two new potential selections within the parameter space where the objective is evaluated. These results are then used to obtain an approximation to the gradient, from which the algorithm proposes two new selections. Video 1 demonstrates an exemplary search of the SPSA algorithms through the parameter space.

#### Objective function

After the presentation of two successive audio–visual stimuli, we estimated brain activation associated with each and compared it to the target brain state: this difference was termed the *loss*. The loss was designed to show whether there was greater activity in the target brain regions for the first or the second combination of audio–visual stimuli. To estimate the loss, we ran separate GLMs on the previous 20 time points (i.e., 40 s: 10 TRs (20 s) covering stimulus presentation and 10 TRs covering subsequent baseline presentation) of the cleaned time courses extracted from the occipital and temporal cortical masks, respectively. Each GLM consisted of an intercept term and two stimulus regressors, which were modeled by convolving a boxcar kernel with a canonical double gamma hemodynamic response function (HRF). This resulted in two estimated regression coefficients per GLM (disregarding the intercept term), representing the first and second stimulus, for which a corresponding post-hoc t-contrast was calculated comparing the two regression coefficients. A positive t-value indicated that the first stimulus presented more closely matched the target brain state while a negative t-value indicated that the second stimulus more closely corresponded to the target brain state. Separate t-values were calculated for the occipital and temporal target brain region. If the two t-values from the different masks were in conflict, the larger t-value was used to determine which of the two states was chosen to govern the next stimulus combination proposed by the algorithm. For half the runs, the loss function was based on maximizing occipital and minimizing superior temporal brain activity, and for the other half this was reversed, as explained above.

#### Stopping criteria

The SPSA algorithm is expected to eventually converge to a local optima as the number of iterations increases (Spall, 1998). In this work, convergence was based on an arbitrary threshold and defined as sampling the same combination of stimuli for three consecutive iterations (i.e., the same stimuli combination was chosen to be optimal on three consecutive iterations). If convergence occurred the scan was stopped. If convergence did not occur within 10 min, the experiment ended automatically in order to keep scanning time to a comfortable length for the participants.

### Bayesian optimization

For Study 2, we employed a Bayesian optimization approach (Brochu et al., 2010b, Snoek et al., 2012). The proposed algorithm consists of an iterative scheme where subjects are presented with an audio–visual stimulus and their current brain state is measured. This information is subsequently provided as feedback and incorporated in real-time to effectively learn the distribution of the latent objective function (in this case the brain activation across target states).

#### Bayesian optimization model

The implementation of a Bayesian optimization algorithm requires two fundamental choices. First, a non-informative prior distribution must be specified for the latent objective function. Here, a Gaussian process (GP) prior is employed due to its flexibility and tractability (Rasmussen and Williams, 2006, Snoek et al., 2012). GPs are fully specified by their mean and covariance functions. Here, a flat (i.e., non-informative) mean function was used. The choice of covariance function when employing GP is fundamental (Brochu et al., 2010b). The squared exponential kernel was used due to its widespread use and simplicity (Rasmussen and Williams, 2006):

$$k\left(x,y\right)=\sigma^{2}\exp\left\{-\frac{{\left(x-y\right)}^{2}}{2l^{2}}\right\}$$

where $x,y\in R^{2}$ correspond to the choice of audio–visual stimuli. The hyper-parameters σ∈R and $l\in R^{2}$ each determine the variance and length scale of the covariance kernel respectively and must be carefully selected. In addition to this covariance function, it is also assumed that observations are corrupted by white noise. This is characterized by constant variance, *σ*_*noise*_^2^. While it is possible to tune these hyper-parameters in real-time, in this work the parameters where selected prior to running the experiments. Formally, the data from Study 1 was employed to tune these parameters using Type-2 maximum likelihood. This corresponds to a computationally feasible approximation where the marginal likelihood is maximized with respect to the hyper-parameters (Rasmussen and Williams, 2006). This is appealing as the marginal likelihood is directly differentiable with respect to the hyper-parameters, yielding computationally efficient estimates. This choice of hyper-parameters was then fixed for all subjects' runs.

#### Acquisition function

Second, it is necessary to specify a function to determine which combination of stimuli to propose next, typically referred to as the acquistion function (Brochu et al., 2010b). Similar to the choice of hyper-parameters, the choice of acquisition function is paramount to the success of Bayesian optimization. Here, we used the expected improvement acquistion function (Brochu et al., 2010b, Srinivas et al., 2010), motivated by recent empirical (Osborne, 2010) and theoretical (Bull, 2011) results. Informally, this choice of acquistion can be seen as trying to maximize the expected improvement (in this case the difference in activation level between the target brain regions, i.e. maximized occipital cortex activity with minimum superior temporal cortex activity) over the current best. An additional attractive aspect of the expected improvement acquisition function is that it has a closed form under a GP prior; allowing the algorithm to rapidly propose a new audio–visual stimulus in real-time.

We define *m*(*x*) as the predictive mean for a point $x\in R^{2}$ and *var*(*x*) as the predictive variance (i.e., *m*(*x*) represents the mean expected difference in brain level activity between the target brain regions for given stimulus *x*, and similarly for *var*(*x*)). The expected improvement is defined as (Brochu et al., 2010b):

$$\mathrm{EI}\left(x\right)=\left(m\left(x\right)-f_{\max}\right)q\left(z\right)+\mathrm{var}\left(x\right)p\left(z\right)$$

where *q*() and *p*() are defined as the cumulative and probability density functions for a standard normal distribution respectively and f_max_ is the maximum observed value of the objective function (Brochu et al., 2010b). Finally, z is defined as:

$$z=\frac{m\left(x\right)-f_{\max}}{\text{var}\left(x\right)}.$$

At every iteration, the next combination of stimuli to be observed is selected by maximizing the expected improvement:

$$x_{\mathrm{next}}=\mathrm{argma}x_{x}\left\{\mathrm{EI}\left(x\right)\right\}.$$

The Bayesian optimization procedure is computationally and mathematically simple. An initial burn-in phase of five randomly selected stimuli is employed. Thereafter, at each iteration, a new audio–visual stimulus combination is proposed by maximizing the expected improvement acquisition function. The proposed stimuli are subsequently presented to the subject and the difference in activation level between the target brain regions is provided as feedback to the algorithm. This feedback is used to update the posterior distribution of the unknown objective function (Rasmussen and Williams, 2006). Due to the properties of GPs, this update is computationally efficient and can be computed in closed form (Brochu et al., 2010b). See Video 2 for an example on how the algorithm is exploring the experiment parameter space over time and learning the relationship between current brain state (i.e., difference in brain activation between the target brain regions) and stimuli combinations.

#### Objective function

After the presentation of a single audio–visual stimulus, we calculated the difference in brain level activation between the two target brain regions. For this purpose, we ran separate GLMs on the previous 10 time points (i.e., 20 s) of the cleaned time courses extracted from the occipital cortex and temporal cortex mask. Each GLM consisted of an intercept term and one stimulus regressor, which was modeled by convolving a boxcar kernel with a canonical double-gamma HRF. We simply took the difference between the resulting regression coefficients and entered them into the Bayesian optimization algorithm.

#### Stopping criteria

Bayesian optimization algorithms are much more sophisticated than the previously used stochastic approximation algorithm (i.e., SPSA). Such algorithms effectively balance a trade-off between exploration and exploitation, which stochastic methods ignore. As a result, it is challenging (and less important) to define a convergence criterion for Bayesian optimization methods. Consequently, each run in this study was terminated after 19 observations had been sampled. This corresponded to 5.26% of the experimental parameter space or 190 TRs (6.3 min).

### Offline analyses on data from Study 1 using Bayesian optimization

In order to explore the potential gain when employing a more sophisticated and model-based optimization method, we conducted offline analyses on the acquired data from Study 1 using the Bayesian optimization approach described above. For this purpose, we re-ran the calculation of the objective function: while for the SPSA algorithm we calculated t-contrasts between two consecutive audio–visual stimuli, the Bayesian optimization approach updates its model estimation after each iteration. Hence, we calculated the difference in brain level activation between the two target brain regions for each observation separately. For this purpose, we ran separate GLMs on the previous 10 time points of the cleaned time courses extracted from the occipital cortex and temporal cortex mask. Each GLM consisted of an intercept term and one stimulus regressor, which was modeled by convolving a boxcar kernel with a canonical double-gamma HRF. We simply took the difference between the resulting regression coefficients and entered them into the Bayesian optimization algorithm. Note that these results are biased as we used data from Study 1 to tune the hyper-parameters of the GP covariance function using Type-2 maximum likelihood; however, this bias does not affect the subsequent real-time data acquired in Study 2.

### Post-hoc whole-brain fMRI analysis

We performed post-hoc fMRI analyses to illustrate that expected patterns of brain activity corresponded to maxima and minima in the experimental parameter space. To do this, we used standard FSL FEAT GLM to determine the effect of the optimal stimuli combinations vs. least optimal stimuli combinations on individual's whole-brain activation. For this purpose we re-ran the Bayesian optimization with all available observations per subject (i.e., aggregating all runs). The resulting model was then used to predict the entire experiment parameter space (361 possible combinations). We identified the optimum of the estimated experiment parameter space as the coordinate with the maximum predicted value by our model. In addition, we identified the coordinate with the minimum predicted value, representing the least optimal stimuli combination for evoking the pre-defined target brain state. Based on these results, we created three regressors that informed the GLM. For the first regressor, we determined all available observations (for determination criteria refer to the Appendix) close to the optimum. For the second regressor we identified all available observations in vicinity to the minimum (see Appendix). Note, that as the real-time stimuli selection using the Bayesian optimization method aims to maximize the expected improvement, the least optimal stimuli combinations were sampled less frequently compared to the most optimal stimuli combinations. All remaining observations were input for the third regressor. An observation was modeled as 10 s long boxcar and subsequently convolved with a double-gamma HRF. In addition, each regressor's first temporal derivative was included in the GLM. This first-level analysis was carried out for each run separately. The resulting parameter estimates of each run were then entered into a higher-level (fixed-effect) cluster-corrected FEAT analysis to summarize the results per individual with respect to the two contrast: ‘most optimal stimuli combinations > least optimal stimuli combinations’ and ‘least optimal stimuli combinations > most optimal stimuli combinations’. All final subject-level images were thresholded using a cluster correction threshold of nominal *z* > 2.3 and a nominal cluster significance threshold of *p* = 0.05. Group-level images were visualized on an average surface brain using MRIcroGL (http://www.mccauslandcenter.sc.edu/mricrogl/). Given that we are contrasting observations classified based on a prior statistical comparison (i.e., the Bayesian optimization results) statistical results should be considered biased. Instead they are presented here for illustrative purposes so the approximate spatial distribution of activation and individual variability can be observed.

### Statistical inference

In order to assess how likely the pattern of results in Study 1 could have occurred by chance, we performed one-tailed non-parametric permutation testing. We created a null-distribution by simulating (10,000 permutations) the mean rate of convergence for 14 runs (7 subjects à 2 runs) when randomly assigning our empirically obtained objective function values (t-values) for each iteration within an experiment. For practical reasons', the maximal rate of convergence was set to 50 — so a *simulated* run that did not converge within 50 iterations was set to the maximal rate. After we obtained a distribution of test values for the mean rate of convergence expected under the null hypothesis, the p-value associated with our observed test statistic was computed. In order to obtain our observed test statistic, we set all *empirical* runs that did not converge or incorrectly converged to 50. This can be considered as a very conservative approach since in reality we stopped a run after 14 iterations (rather than 50). A more liberal approach for obtaining our test statistic would be to set all runs that did not or faulty converged to e.g. the mean of the null distribution instead (i.e., 27.4). We also report results when repeating the same procedure for the median rate of convergence.

In order to assess the statistical significance of our group-level results obtained when using only the first run of each subject in Study 2, we again performed one-tailed non-parametric permutation testing. For this purpose, we created a null-distribution by simulating the Euclidean distance of the estimated optimum from the hypothesized optimum when shuffling our empirically obtained objective function values (difference between the regression coefficients at each observation). The final model after 95 iterations (5 subjects à 19 iterations) was then used to predict the entire experiment parameter space. Finally, the Euclidean distance of the predicted optimum from the hypothesized optimum was calculated. This simulation was run for 10,000 permutations in order to obtain a distribution of test values expected under the null-hypothesis.

### Contrast-to-noise ratio

In order to provide estimates of how different levels of signal and noise affect the Bayesian optimization, we performed supporting analyses involving simulations and further interrogation of our empirical data. While SNR measurements can be very useful to assess data quality with respect to scanner hardware or scanning sequence, contrast-to-noise ratio (CNR) is a measure of the detectability of the contrast of interest (Welvaert and Rosseel, 2013). According to a recent review (Welvaert and Rosseel, 2013) CNR values reported in the literature varied from 0.5 to 1.8; therefore, we performed simulations with CNR values between 0.1 and 1.8. In addition, we computed the CNR of our empirical data for each subject separately.

#### Simulations

The “ground truth” for the simulated contrast of interest was modeled according to the hypothesized shape from our actual experiment: with an audio–visual stimulus that maximally evokes target neural activity in the center of the grid and the least optimal stimuli combinations in each of the grid's corners with smooth transitions in-between (see Fig. 6a). For computing CNR, we calculated the amplitude of the signal divided by the standard deviation of the noise (Welvaert and Rosseel, 2013). Since we had 361 different signal amplitudes, we took the mean of all (absolute) amplitude values (absolute values were taken, because the simulated contrast could be either positive or negative, for different locations in the parameter space). According to the range of simulated CNR values reported above, the standard deviation of Gaussian noise varied between 6.06 and 0.34 while the mean was zero.

For each CNR value tested, we ran 100 simulations. The maximum number of iterations was set to 100 with the first five (randomly selected) observations serving as burn-in phase (identical to our actual experiment). Thereafter, at each iteration, a new audio–visual stimulus combination was proposed by maximizing the EI acquisition function and sampled by the Bayesian optimization algorithm in the next iteration. At each iteration, we identified the predicted optimum as the location in the parameter space that maximized the predicted value of the objective function. As a measure of accuracy of these predictions at each iteration, we calculated the Euclidean distance between the predicted and modeled optima (at coordinate [10 10]). We also computed a spatial correlation between the algorithm's predictions for the whole parameter space and the “ground truth” parameter space, assessing how well the algorithm estimates not just the optimum but also the whole space.

As reported above, the choice of hyper-parameters of the covariance function is critical for the success of the Bayesian optimization. Identical to our actual experiment, we selected these parameters before running the 100 simulations for each CNR value. For this purpose, we randomly selected 50 observations and used these independent observations to tune the hyper-parameters using Type-2 maximum likelihood (Rasmussen and Williams, 2006). This was done for each simulated CNR value separately and the hyper-parameters were then kept fixed for all 100 simulations.

For each observation at any stage of the simulation (including tuning of hyper-parameters and burn-in phase), we added a random sample from a Gaussian noise distribution (with varied standard deviation depending on the CNR simulated) to the amplitude of the signal before it entered the Bayesian optimization algorithm.

#### Empirical CNR

For each run of each subject, we calculated the CNR according to the definition above: we took the mean of all (absolute) signal amplitudes (i.e., difference between the resulting regression coefficients) and divided it by the mean standard deviation of the time series derived from the visual and auditory cortex target brain areas.

## Results

### Converging to hypothesized optimal stimuli combination

In Study 1, each subject underwent two closed-loop runs that only differed in the a-priori defined target brain state (Fig. 2b). Across all seven subjects, the SPSA algorithm correctly converged to the targeted optimal audio–visual stimulus in 11 out of 14 runs (Table 1) within 10 min. For one run (sub_03), the SPSA faulty converged slightly off the optimum (at [4 10] instead of [1 10]) while for two other runs, convergence did not occur within 10 min. In one of the runs where convergence did not occur (sub_04), the algorithm remained in the vicinity of the hypothesized optima, which was visited more frequently than any other stimulus combination. We performed conservative permutation testing (i.e., setting the three runs that did not converge to 50) and found the probability of our empirical mean rate of convergence of 16.86 occurring by chance was *p* = .0079. Using a less conservative approach (i.e., setting all runs that did not converge to the mean of the null distribution instead (27.4), see Methods section) resulted in an empirical mean rate of convergence of 12.01 and lead to a more significant result (*p* = .00021). When repeating the same procedure for the median rate of convergence, we obtain for both, the conservative and liberal approach, the same test statistic, i.e. a median rate of convergence of 9, corresponding to *p* = .016. Although SPSA accurately and efficiently found the hypothesized optimal audio–visual stimulus, it provides a very limited understanding of the underlying relationship between stimuli and neural responses as outlined in the Introduction section.

### Mapping the underlying relationship between stimuli and neural responses

In order to explore the potential gain when employing a more sophisticated optimization algorithm instead, we conducted offline analyses on the acquired data from Study 1 using the Bayesian optimization approach described above. We found that for even very few observations, the Bayesian optimization accurately mapped out the hypothesized stimuli–response relationship for each run in every subject (Supplementary Fig. 2). While these results relied on the available observations that were proposed by the SPSA in real-time, the superiority of the Bayesian optimization could only be validated in another real-time experiment. For this purpose, we conducted Study 2 in which the algorithm needed to traverse an even larger (and hence, more challenging) experiment parameter space (Fig. 2c).

**Fig. 3:**
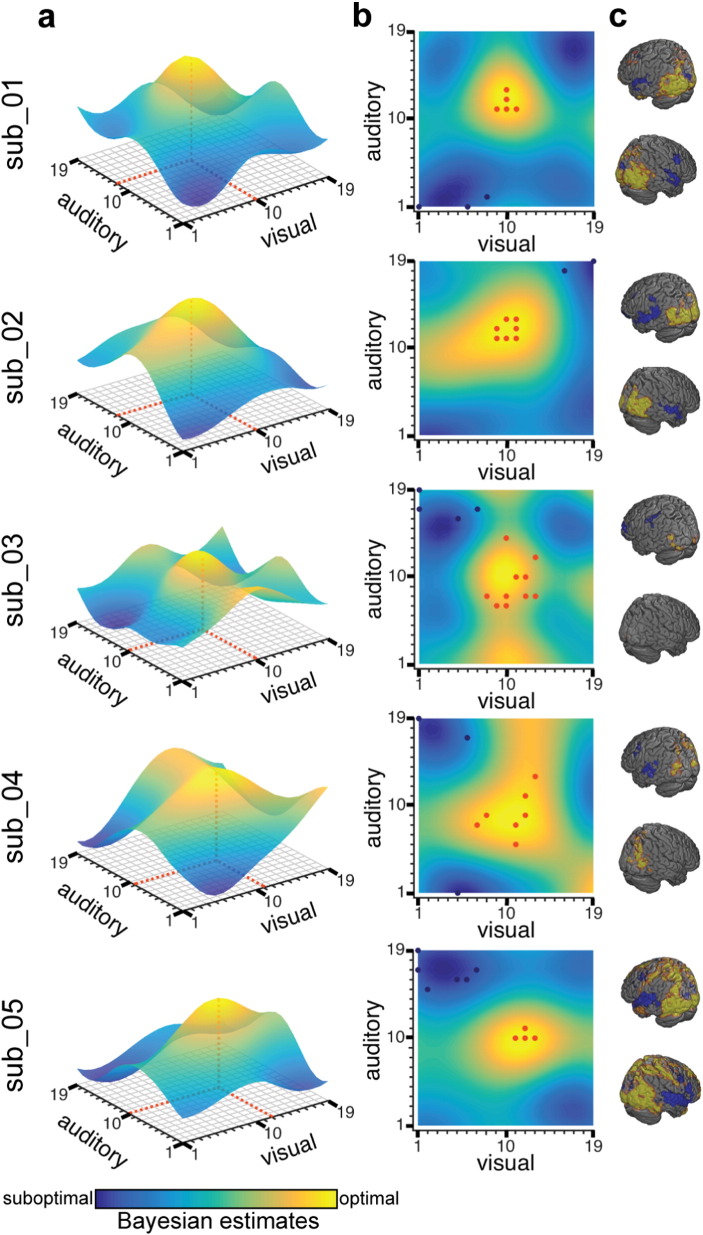
Mapping between stimuli and neural responses using the Bayesian optimization approach. (a and b) Experiment parameter space estimates for each subject (sub_), taking all available runs into account. The color bar represents the estimates by the Bayesian method on how optimal the experimental condition is for evoking the target brain state: the higher the predicted value, the more optimal the stimuli combination (yellow); the lower the predicted value, the less optimal the stimuli combination (dark blue). The Bayesian optimization accurately recovers the hypothesized relationship between stimuli and neural responses: with optimal stimuli combinations in the center of the grid and least optimal stimuli combinations in each of the grid's corners. To facilitate visual inspection, the exact coordinate of the empirical optimum (i.e., maximum predicted value) is marked as red dashed line. (c) Post-hoc fMRI pattern of activation. The orange dots in (b) were entered into the general linear model (GLM) as observations for the regressor modeling the most optimal stimuli combinations while the dark blue dots entered the GLM as least optimal stimuli combinations. The cluster corrected results of the higher-level (summarized over all runs) analysis are shown. The contrast ‘most optimal stimuli combinations > least optimal stimuli combinations’ is shown in yellow while the contrast ‘least optimal stimuli combinations > most optimal stimuli combinations’ is rendered in dark blue. The data summarize results from all four runs for all individuals except for sub_04 who only completed a single run due to MRI technical failure during scanning.

In Study 2, the Bayesian optimization method found the optimum close to the targeted optimal audio–visual stimulus combination (which was located at coordinate [10 10], Fig. 2c) for all five participants when taking all runs into account (Fig. 3a–b). The most optimal audio–visual stimuli were found to be in the center of the grid (i.e., maximum predicted values, shown in yellow) and the least optimal stimuli in each of the grid's corners (i.e., minimum predicted values, shown in dark blue). We identified the empirical optimum as the coordinate that maximized the predicted value (illustrated as red dashed line in Fig. 3a) under the learned representation of each subjects' response. Across all subjects, the mean ± SD Euclidean distance between the empirical optimum and the hypothesized optimum was 1.48 ± 0.87 (visual: 0.6 ± 0.89, auditory: 1 ± 1). In addition, a post-hoc whole-brain fMRI analysis was performed on this data (Fig. 3c), which showed that the target brain regions (lateral occipital cortex and superior temporal cortex) were well suited for capturing the desired effects. This was expected as we selected these brain regions based on previous work (Braga et al., 2013) because they strongly activate for complex visual or auditory stimuli, respectively. This analysis thus serves to validate that our approach is picking up neurally and cognitively meaningful differences between the target brain regions. Furthermore, this unconstrained whole-brain analysis provides insight about additional brain regions that map similarly onto the experiment parameter space as the target brain regions. An example is the early visual cortices (V1/V2), which were not included in the target occipital mask.

Most importantly, we observe some inter-individual differences. While the Bayesian optimization algorithm converged to a relatively confined representation of the parameter space with a clear peak in the center of the grid for subjects sub_01, sub_02 and sub_05, the estimated parameter space for sub_03 appears much more distributed and less clear. Although, the algorithm correctly found the optimum to be located exactly at the hypothesized optimum (i.e., [10 10]), the available observations of this subject around the optimum are actually more widespread compared to other subjects.

### Assessing the algorithm's performance in real-time

**Fig. 4:**
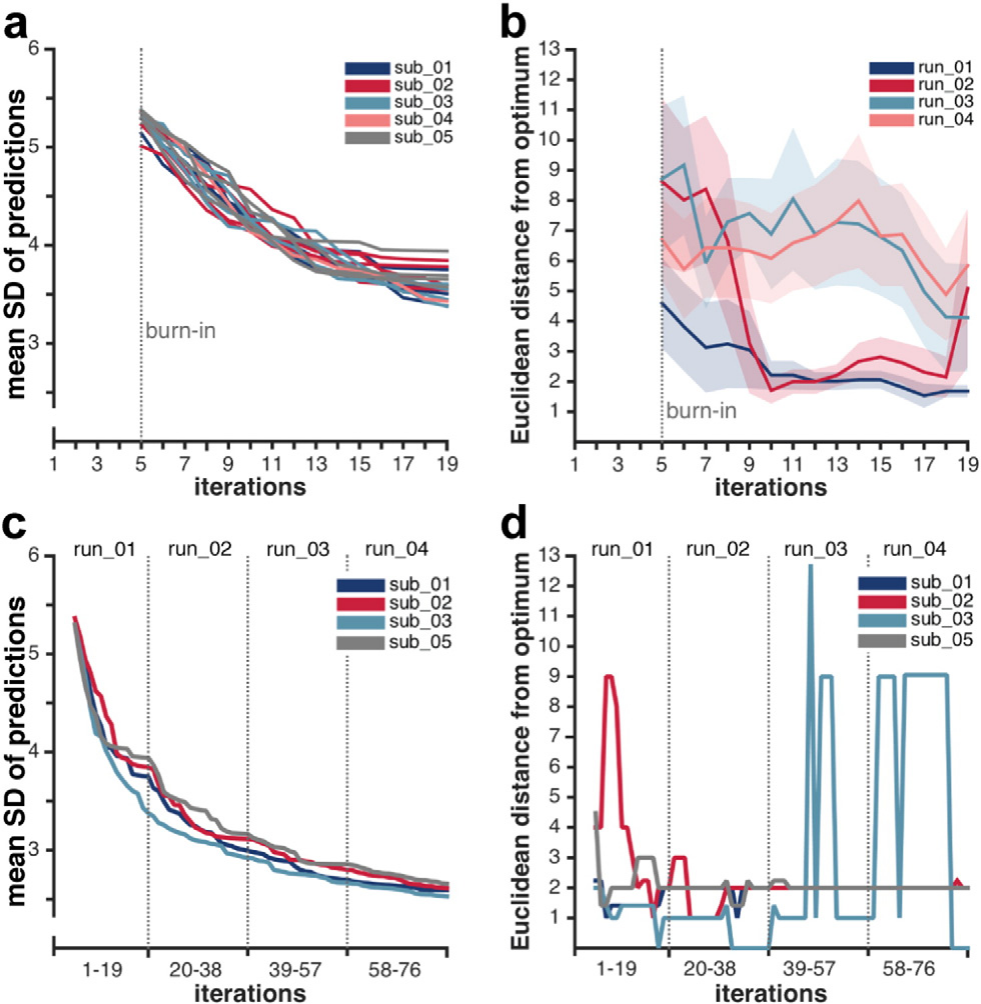
Summary measures of Bayesian model performance. As the first five iterations were used as a burn-in for a first estimate of the Bayesian model, they are not depicted here. (a–b) Performance measures were derived at each iteration for each run by updating the model with every new observation made, i.e. incrementally re-simulating the online scenario. (c–d) Performance measures were derived when updating the model derived from the first run with each new observation made in succeeding runs from the same subject. Results are shown for four subjects as only one run was available for subject sub_04. (a) Mean SD across the predicted values of all possible 361 audio–visual stimulus combinations for each run of every subject. Each color represents a single subject. (b) Mean Euclidean distance between predicted optimum (coordinate with maximum predicted value) from hypothesized optimum (i.e., [10 10]) across all subjects for each run. Each color represents a different run. Shaded areas represent the SEM. (c) Mean SD across predicted values of all possible stimuli combinations at each iteration for concatenated runs. (d) Euclidean distance of predicted optimum from hypothesized optimum at each iteration for concatenated runs. Each color represents a single subject. Note, that at the final update (at iteration 76), the results are the same as those depicted in Fig. 3a–b.

Further, we assessed the real-time (rather than aggregate) performance of the Bayesian optimization approach for each run separately to judge how new information updates the model. Fig. 4a depicts the mean SD of each run for all subjects at every iteration. As expected, uncertainty of our predictions decreased with an increasing number of observations, thus demonstrating that the algorithm is learning in real-time. Inter- and intra-individual differences in the rate of change of the algorithm's uncertainty seem negligible. Further, we found that the level of uncertainty stabilizes towards the end of the run and little to no additional decrease in uncertainty is evident with more observations. Next, we calculated the Euclidean distance of the empirical optimum from the hypothesized optimum for every model update after each observation. Given that there was considerable variance across the runs for each subject (Supplementary Fig. 3), we analyzed each run separately (averaging across all subjects) (Fig. 4b). Interestingly, the first and second run outperformed the last two runs with respect to a smaller mean Euclidean distance from the hypothesized optimum as well as smaller SEM towards the end of these runs. This finding may relate to habituation to the audio–visual stimuli and/or enhanced tiredness/boredom on the part of the subjects in the last two runs.

### Assessing how many observations and runs are needed

In a subsequent analysis, we assessed how the Bayesian model estimate evolves over time, when not treating each run independently, but rather updating the model from the first run with each new observation made in succeeding runs from the same subject. In this way, the model estimated following the first run was used as the prior model for the second run, and so on. Note that Bayesian optimization is particularly well suited to such an approach. We observed that there is a rapid learning gradient, i.e. decreased uncertainty, over the first run while it levels off for the last two runs (Fig. 4c). Further, we found that for all subjects the minimum Euclidean distance to the hypothesized optimum was obtained at the end of the first run or over the course of the second run (Fig. 4d). Note that a large Euclidean distance in the beginning of the first run is not disadvantageous as the algorithm is still exploring the parameter space at this stage (as the case for subject sub_02). While for three subjects, no further optimization was achieved when adding observations made in the second, third and fourth run; we even find an adverse effect for one subject (sub_03) in the last two runs. These results also neatly correspond to our findings with respect to sub_03 mentioned above (Fig. 3a–c). The last two runs for this subject seemed corrupted by very noisy measurements that lead to sudden changes in the algorithm's experimental parameter estimates. Based on these results, we concluded that there was relatively little or no gain (in terms of reduction of uncertainty or distance from hypothesized optimum) from adding a second, third or fourth run to our experiment.

**Fig. 5:**
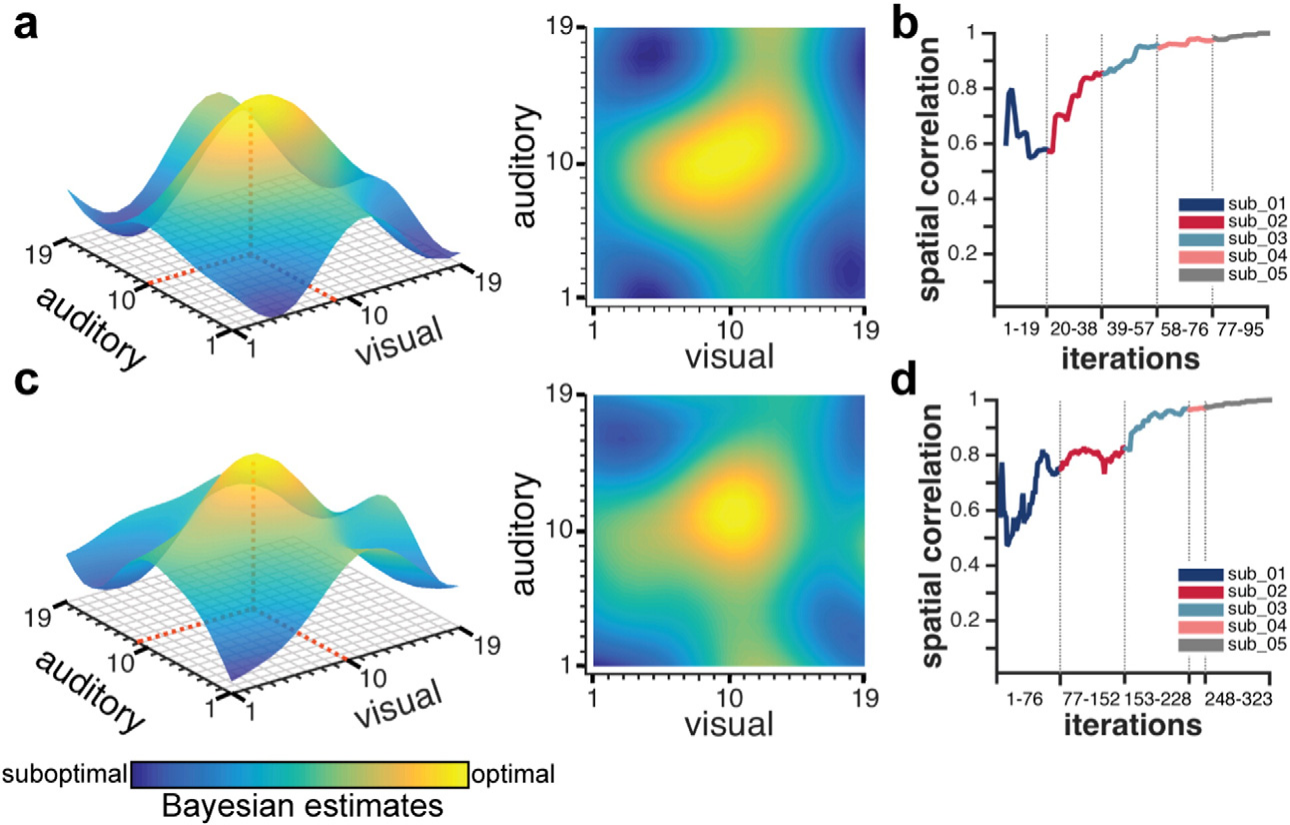
Comparison of parameter space estimation based on different number of runs from all subjects. (a) Parameter space estimates obtained when concatenating only the first runs of all subjects. The color bar represents the estimates by the Bayesian method on how optimal the experimental condition is for evoking the target brain state: the higher the predicted value, the more optimal the stimuli combination (yellow); the lower the predicted value, the less optimal the stimuli combination (dark blue). To facilitate visual inspection, the exact coordinate of the empirical optimum (maximum predicted value) is illustrated as red dashed line. (b) Spatial correlation between the final estimated parameter space at iteration 95 and each previous observation (from iteration 5 on). Different colors represent observations from different subjects that were used for the Bayesian model update. (c) As comparison to (a), parameter space estimates obtained when concatenating all available runs of all subjects. (d) Spatial correlation between the final estimated parameter space at iteration 323 and each previous observation (from iteration 5 on). Different colors represent observations from different subjects that were used for the Bayesian model update.

This finding is also emphasized when estimating the experiment parameter space based only on the first runs of each subject. Using this approach, the model converged to a predicted optimum at coordinate [9 10] (Fig. 5a); corresponding to a Euclidean distance of 1. This finding was identical to the minimum Euclidean distance when using all available runs of all subjects (Fig. 5c). In this case, the predicted optimum was located at the coordinate [10 11]. Also when quantitatively assessing the similarity between the estimated parameter spaces based on only the first runs vs. all runs of all subjects, we find a very high spatial correlation (*r* = .92). Furthermore, for both estimated parameter spaces separately, we computed the spatial correlation between the final estimation and each previous observation. The results of these analyses are depicted in Fig. 5b and d, respectively. We find that, for both models, the similarity to the final parameter space estimates consistently increases when including observations from three subjects with only marginal improvements for subsequent subjects. In order to assess the statistical significance of our group-level results obtained when using only the first run of each subject, we performed permutation testing. When testing our empirically obtained Euclidean distance of 1 from the optimum against the null distribution, we obtained *p* = .00022.

### Assessing how contrast-to-noise ratio is affecting the optimization

**Fig. 6:**
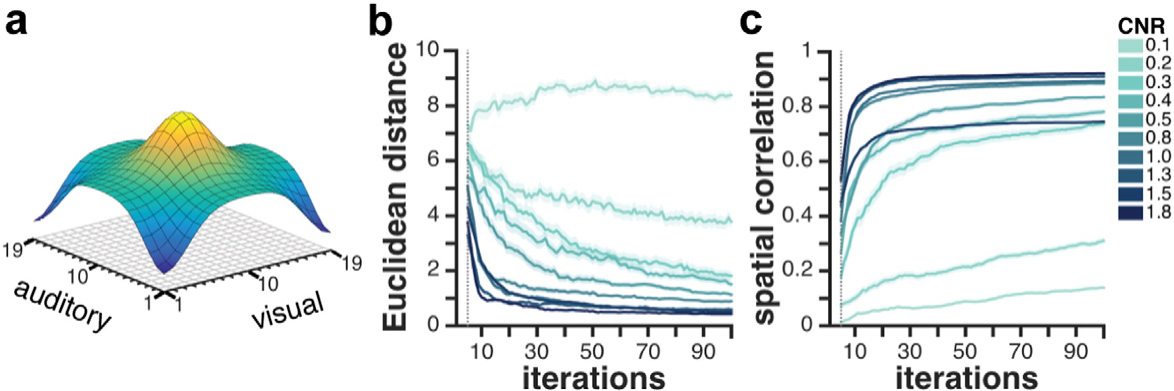
Results of CNR simulation analyses. (a) Modeled objective function (“ground truth”) used for simulations. (b) Mean ± SEM (shaded areas) Euclidean distance (across 100 simulations) between predicted optimum and modeled optimum at each iteration for different CNR values. (c) Mean ± SEM (shaded areas) spatial correlation (across 100 simulations) between the algorithm's predictions for the whole parameter space and “ground truth” parameter space at each iteration for different CNR values. As the first five iterations were used as a burn-in for a first estimate of the Bayesian model, they are not depicted here (gray dashed line). Simulations were performed for 10 different CNR values, ranging from 0.1 (bright blue) to 1.8 (dark blue).

Supporting simulation analyses were performed to assess the influence of CNR on the accuracy and efficiency of the Bayesian optimization. For a range of CNR values we performed 100 simulations over 100 iterations and computed the Euclidean distance between the predicted and modeled optima as well as spatial correlation between the predicted and modeled parameter space. The mean ± SEM (across 100 simulations) Euclidean distance and spatial correlation results are depicted in Fig. 6b and c, respectively. We found that for typical CNR values reported in the literature (0.5–1.8), the accuracy of prediction (in terms of Euclidean distance and spatial correlation) of the Bayesian optimization improved over time. Interestingly, for CNRs above 0.8, high accuracy is achieved after only a few observations (between 12 and 20) with little improvement over more iterations. Although a constant improvement in prediction accuracy for higher CNR values could be observed, the difference in accuracy for CNR values from 0.8 on seems relatively small. In contrast, for low CNRs, such as in the range between 0.2 to 0.5 more observations seem beneficial for the success of the Bayesian optimization. We note though that even for low CNR values, we can achieve satisfactory accuracy after a realistic and feasible number of iterations: for example for a CNR of 0.3, after 50 iterations (50 × 20 s corresponds to approximately 16 min) the mean Euclidean distance between predicted and modeled optima is around 3 and the spatial correlation between the predicted and modeled parameter space is close to 0.7. Importantly, for signals that are corrupted by high amounts of Gaussian noise, as is the case for CNR of 0.1, the Bayesian optimization fails to map out the underlying objective function.

In addition to the simulations, we also calculated the CNR of each subject in our actual experiment. Across all runs, we found high mean ± SD CNR values for sub_01 (1.60 ± 0.03), sub_02 (1.18 ± 0.29) and sub_05 (1.33 ± 0.92). The lowest CNR value was found for sub_04 but only one run was performed (0.49). In comparison to the subjects that performed all runs, sub_03 exhibited the lowest CNR (0.73 ± 0.20).

## Discussion

The present work demonstrates the feasibility of our proposed *Automatic Neuroscientist* approach, turning on its head how a typical fMRI experiment is carried out. The results from Study 1 show that the approach can rapidly and accurately adjust the experimental conditions in real-time in order to maximize similarity with a target pattern of brain activity. We achieved convergence for the hypothesized optimal audio–visual stimulus combination in 11 out of 14 runs in less than 10 min. In Study 2, by employing a Bayesian optimization approach we demonstrated that we could rapidly obtain an accurate estimation of the whole experimental parameter space in each individual. Moreover, we showed that using only the first run of three subjects produced a reliable solution at a group-level. In comparison, if we had sampled each of the 100 possible states in Study 1, the scanning time would have been a minimum of half an hour per subject (100 states × 20 s). For Study 2, each run only lasted 6.3 min, while it would have taken more than two hours scanning time for each individual to exhaustively test all combinations in the parameter space (361 states × 20 s). This framework, therefore, provides us with an efficient and novel way of using fMRI to explore the relationship between tasks and the brain, with a number of different, potential applications.

In the following paragraphs we describe four different potential scenarios where the *Automatic Neuroscientist* would be useful.

### Scenario 1: opening new avenues in cognitive neuroscience

While the current study demonstrates the feasibility of our approach using perceptual stimuli, it is eventually aimed to higher-level cognitive tasks. Our method provides a novel tool to address the other side of the many tasks to many regions mapping by understanding how many different cognitive tasks can activate the same brain system, thereby complementing meta-analytic approaches. Our approach also strives to capture individual differences in a different way, not in terms of across-individual variability in regional activation for a given task, but in terms of how a set of tasks/stimuli relate to activity in that region.

### Scenario 2: tailoring clinical rehabilitation therapy to the patient

#### Rehabilitative cognitive or behavioral therapy

Our approach could be useful in tailoring cognitive or behavioral rehabilitation to a specific patient's needs. For instance, altered connectivity between the default mode network and fronto-parietal networks during effortful cognitive tasks has been related to cognitive impairment in patients suffering from traumatic brain injury (Jilka et al., 2014). Therefore, some type of computerized behavioral intervention can be optimized (i.e., parameters such as difficulty, inter-stimulus interval, task type) and tailored to individual patients, to develop a cognitive-behavioral training regime designed to normalize dysfunctional connectivity. This tailored computer training could then be extensively delivered to the patient outside the scanner (e.g., at home), and potentially, improve cognitive function. In line with this application scenario, advancing personalized interventions by means of real-time fMRI has been established as the overall goal of the fMRI neurofeedback community (Stoeckel et al., 2014).

#### Brain stimulation

Studies involving non-invasive brain stimulation have reported promising modulation on cognitive and behavioral performance (Kuo and Nitsche, 2012, Mondino et al., 2014). Simultaneous fMRI is increasingly used to understand the functional brain networks affected by the intervention (Antal et al., 2011, Saiote et al., 2013, Sehm et al., 2012). In most studies only a small range of possible stimulation parameters is varied and investigated. However, the optimal stimulation paradigm may vary across individual patients based on the pathology of the disease. We propose to tackle this issue by combining our approach with short periods of brain stimulation in the scanner and systematically explore a variety of stimulation parameters (including stimulation site, intensity, duration, amplitude and phase of the stimulation) aimed at maximizing ‘healthy’ patterns of brain activity for a specific patient. The great potential of personalized stimulation therapy was recently highlighted using deep brain stimulation (Little et al., 2013).

### Scenario 3: optimize stimuli in fMRI piloting

Our approach also has practical potential benefits, when designing fMRI experiments. Using our framework, pilot scans can be conducted in a more efficient and principled way, to find the set of e.g., stimuli/task parameters that maximally evoke the target brain response, before acquiring larger datasets, potentially dramatically improving the contrast-to-noise ratio of resulting experiments.

### Scenario 4: assessing an individual's preferences

Another potential application of our proposed framework is to assess subjects' preferences for specific types of stimuli in an efficient way, and to design stimuli that maximally activate brain regions known to be involved in, e.g., reward. For example, stimulus features could be systematically explored and optimized towards a desired response in the brain when designing an advertisement.

Importantly, the choice of optimization algorithm will depend on the specific scenario the *Automatic Neuroscientist* will be applied to. For example, when optimizing stimuli in fMRI piloting (Scenario 3) or assessing an individual's preferences (Scenario 4), one might only be interested in rapidly converging to the best stimuli parameters. In such cases, the application of simple model-free algorithms, such as the SPSA may be sufficient. This class of optimization algorithms is purely exploitative as it is based on a stochastic approximation to gradient ascent. The advantage is that it does not require prior empirical data to tune e.g. the hyper-parameters of the covariance function, as it is the case for the Bayesian optimization approach presented here. However, when the research question shifts to gain an understanding of the underlying relationship between stimuli and neural responses across the whole parameter space such as described in Scenario 1 and Scenario 2, more complex optimization algorithms are required. In particular, using a Bayesian optimization scheme, the balance between exploration and exploitation can be determined by the choice of acquisition function. Throughout this work, we have employed an acquisition function based on the Expected improvement (EI). While this acquisition is well documented for looking to balance the exploration-exploitation trade-off, other more exploitative acquisition functions (e.g., the probability of improvement acquisition, (Brochu et al., 2010b)) could be applied depending on the aim of the study. For very simple experiment parameter spaces (such as in Study 1 for example) it would be possible to estimate the parameter space without real-time optimization, e.g., based on the repetitive presentation of a random subset of stimuli in line with ‘classical’ work on event-related designs (Friston et al., 1999, Josephs and Henson, 1999), and then estimating optimal values for auditory and visual dimensions offline. However, such approaches cannot easily or efficiently be extended to more complex experiment parameter spaces spanning over multiple dimensions and involving more complex underlying stimulus–response-relationships (e.g., multiple optima). Due to the ‘curse of dimensionality’ (Bellman, 1957), sampling from such spaces would require an exponential number of samples, underlining the advantage of the data-driven trade-off between exploration and exploitation in the real-time optimization approach.

Furthermore, depending on the research question, the reliable mapping of an individual or a group's parameter space will be of primary interest. As reported above, the former can be accurately achieved by conducting multiple runs for an individual and estimating the stimuli-responses mapping based on all observations for that subject. However in the latter case, fast and reliable results are likely to be obtained from estimating the parameter space based on observations from multiple subjects while the number of runs per individual is likely to be less critical, as described in our findings above. We note, that in the work presented here, we employed a simplified way of combining observations across subjects. While this approach can be justified with regard to well-documented similarities in subjects' responses to the perceptual stimuli (Braga et al., 2013) tested here, a more rigorous approach to be explored in future work would involve combining observations across subjects employing a hierarchical GP (Hensman et al., 2013).

Within the field of Bayesian optimization, the choice of hyper-parameters of the covariance functions has received some attention. While we selected the parameter prior to running the experiment and kept them fixed for all subjects, it is equally possible to tune the hyper-parameters in real-time for each subject or each run independently. Others have proposed learning the hyper-parameters via the introduction of a particle filter (Brochu et al., 2010a). Again, this will depend on the specific scenario the *Automatic Neuroscientist* will be applied to and if high inter-subject variability in CNR is expected or is the aim of the study.

In order to address the question of generalizability of the framework to other paradigms (with different contrast-to-noise levels), we performed supporting simulation analyses. We demonstrated that the accuracy and efficiency of estimating the experiment parameter space varies as a function of CNR. The CNR will be influenced by the complexity of the parameter space and the specific paradigm used. We acknowledge that for more subtle distinctions between relatively similar cognitive tasks, the CNR is expected to drop and hence, the algorithm is expected to take longer and will be less accurate to estimate the parameter space on a single-run level. However, we showed that even for low CNR values (down to 0.2), the Bayesian optimization achieved satisfactory accuracy after a realistic and feasible number of iterations. When taking a look at our empirical CNR values we found inter-individual differences, with relatively low CNR for subject sub_03. Here, it should be noted that we did not account for autocorrelations when calculating the standard deviation from the empirical fMRI time-series, hence our CNR values might be biased (Woolrich et al., 2001). Interestingly though, sub_03 is the same subject for which the Bayesian optimization converged to a less confined representation of the experiment parameter space when aggregating all available runs (Fig. 3a–b). This suggests that for sub_03, there may have been more noise present, and as a consequence the algorithm was less certain about the location of the optimum and so sampled more varied points in the parameter space. This is also reflected in the whole-brain fMRI results, which clearly showed weaker activations than for the other subjects (Fig. 3c). Importantly, although the algorithm was clearly exposed to very noisy and/or weaker differences in brain activation, it correctly converged to the optimum when taking all runs into account from this subject. This finding demonstrates that the Bayesian optimization is relatively robust to low CNRs when aggregating observations from multiple runs. Furthermore, CNR could be boosted by increasing block length and number of observations per run in low CNR scenarios.

For the *Automatic Neuroscientist* to become a useful tool for the potential applications described above, there are a number of areas for ongoing and future work.

One avenue for future work is developing online stopping criteria; i.e. automatically ending the current run as soon as the uncertainty of the algorithm over the parameter space is sufficiently small. Reducing the scanning time is particularly crucial in the context of fMRI experiments in light of the high imaging costs involved and limited attentional capacities of subjects. Feng et al. (2015) have recently developed a sequential probability ratio test as well as decision rules for stopping stimulus administration when sufficient statistical evidence is collected. Within the context of Bayesian optimization, there is limited work studying stopping criteria as the focus has traditionally been on online learning (Hoffman et al., 2014). We have made a first step in this direction by proposing two stopping criteria for Bayesian optimization methods that we empirically validated using data from Study 2 presented here (Lorenz et al., 2016). The development of online stopping criteria is of paramount importance when the *Automatic Neuroscientist* is applied to clinical populations that are characterized by impaired attention (e.g., attention deficit disorder (Stins et al., 2005), traumatic brain injury (Whyte et al., 1995), or bipolar disorder (Clark et al., 2002)), as well as when children or the elderly are studied.

Another challenge will be the parameterization of the experiment space. We suggest that a range of methods can be used to define the experiment parameter space, depending, in part, on the rationale for the experiment. Firstly, for certain paradigms, the dimensions of the experiment parameter space are very clear, corresponding to intrinsically monotonically increasing/decreasing values of interest to the experimenter. Examples would be: finding the optimal task difficulty, inter-stimulus interval, stimulus duration, drug dosage as well as amplitude, frequency and phase parameters for non-invasive brain stimulation. Secondly, behavioral data could be used to parameterize the experiment space for more complex cognitive tasks, on the assumption that neural organization may, to a first approximation, reflect behavior. For example, Hampshire et al. (2012) performed principal component analysis (PCA) on behavioral data of 12 different cognitive tasks from a large population (n = 44,600). They identified two components corresponding to the psychological constructs working memory and reasoning, with each of the 12 tasks loading to a different degree on them. Interestingly, when repeating the same 12 tasks in the MRI scanner with 16 participants and performing PCA on the imaging data, they identified two distinct functional brain networks that show highly similar task loadings to the two constructs identified by behavioral data. This study demonstrates that data dimensionality reduction techniques such as PCA on behavioral data could be used to define neurally meaningful experimental parameter spaces for higher-level cognitive tasks. The components identified could define the dimensions of the experimental parameter spaces with the tasks aligned along those dimensions according to their loading on the corresponding component. Thirdly, prior meta-analysis of functional neuroimaging data could be employed to extract sets of tasks that are have been associated with a given target brain region. An example would be to build upon work by Yeo et al. (2014) in which a set of cognitive components was identified using a hierarchical Bayesian model based on 10,449 experimental contrasts covering 83 BrainMap-defined task categories. Importantly, for each cognitive component they also made publicly available the probability of each task recruiting those components. Again as explained above, the cognitive component could resemble the dimensions of the parameter space with the tasks sorted according to their probability of recruiting them. Finally, future work could also focus on estimating and refining the parameter space in real time, as part of the optimization process itself.

Adaptively optimizing experiments in a closed-loop manner have been studied in the field of neurophysiology for decades. Those studies usually focus on finding the “preferred stimulus” or determining the receptive field of sensory neurons, such as in the cat's visual cortex (Jones and Palmer, 1987a, Jones and Palmer, 1987b). The preferred stimulus is usually defined as the stimulus that maximizes a neuron's firing rate (Chambers et al., 2014, Nelken et al., 1994, O'Connor et al., 2005). Typically, model-free methods are employed that measure the gradient of an objective function with respect to a set of perturbed versions of the original stimulus (Edin et al., 2004, Földiák, 2001, Machens et al., 2005). Others propose a sequential design in which the posterior distribution is modified after each observation and this update is used to inform the selection of the next stimulus (Benda et al., 2007, Lewi et al., 2008). While the former is similar to the model-free SPSA approach in Study 1 presented here, the latter can be qualitatively compared to the Bayesian optimization approach employed in Study 2.

In a similar vein to the work we present, are adaptive brain–machine interfaces using real-time fMRI. Gantner et al. (2010) modified the transparency of an image of a house depending on the activity in a ‘house’ processing brain area. Another recent closed-loop neurofeedback study convincingly demonstrated how performance in a sustained attention task could be improved by increasing task difficulty (i.e., mixture of composite stimuli) as soon as attentional lapses were detected (deBettencourt et al., 2015). Similarly, Feng et al. (2015) addressed the possibility of dynamically adjusting task difficulty levels in order to determine the minimum task difficulty level that will activate a given brain area but only simulations have been carried out. To our knowledge only one study has applied real-time fMRI to automatically and efficiently search through a large set of possible stimuli. In this study, Cusack et al. (2012) employed online multivariate pattern analysis to converge to a subset of images that evoke a similar brain activation pattern than a reference image. This subset of images was then referred to as the references image's “neural neighborhood”. While this study is in line with the early neurophysiological work, it does not provide a generalizable framework that is applicable to numerous different research questions.

With the work we present here, we aim to stimulate the field, to explore the wide range of novel applications involving closed-loop real-time fMRI. We envision that the framework explained here, will be added to the standard toolkit of modern functional imaging.

The following are the supplementary data related to this article.

Supplementary material

Video 1 The video shows how the stochastic approximation is performed using the SPSA algorithm. At each iteration, the SPSA randomly proposed two new audio–visual stimulus combinations within the parameter space where the objective is evaluated. These results are then used to obtain an approximation to the gradient, from which the algorithm proposes two new stimuli selections. This cycle continues until some stopping criterion is reached. In our work, convergence was based on an arbitrary threshold and defined as sampling the same combination of stimuli for three consecutive iterations (i.e., the same stimuli combination was chosen to be optimal on three consecutive iterations). If convergence occurred the scan was stopped. If convergence did not occur within 10 min, the experiment ended automatically in order to keep scanning time to a comfortable length for the participants.

Video 2 The video shows how the Bayesian optimization algorithm is exploring the experiment parameter space over time and is learning the relationship between target brain state and audio–visual stimuli combinations over different iterations/observations. The height of the objective function represents the predictions by the Bayesian method on how optimal the experimental condition is for evoking the target brain state: the higher the predicted value, the more optimal the stimuli combination (yellow); the lower the predicted value, the less optimal the stimuli combination (dark blue). As can be seen, from iteration 12 on the algorithm seems to have obtained a global understanding of the experiment parameters space and keeps sampling the predicted optimum over multiple iterations, hence trying to maximize the expected improvement as described in the Methods section. Each run was automatically stopped after 19 observations.

## Figures and Tables

**Table 1:** Convergence results of all subjects in Study 1 for both runs.

Subject	Target brain state (1) (iterations/min)	Target brain state (2) (iterations/min)
sub_01	10/6.67 min	9/6 min
sub_02	13/8.67 min	8/5.33 min
sub_03	7/4.67 min	*Faulty convergence*
sub_04	9/6 min	*No convergence*
sub_05	6/4 min	10/6.67 min
sub_06	6/4 min	4/2.67 min
sub_07	*No convergence*	4/2.67 min

## Appendix Determination criteria for entering observations as regressors for post-hoc whole-brain fMRI analysis

For determining which observations were entered as regressors for the post-hoc whole-brain fMRI analysis, the challenge was to keep the number of observations and the Euclidean distance for labeling observations as optimal/least optimal approximately consistent across subjects. To this end, the following heuristics were used:

*Criteria for the regressor modeling the most optimal stimuli combinations.* For three subjects (sub_01, sub_02, sub_05) we chose a Euclidean distance ≤ 1.5 corresponding to 13, 12 and 17 observations, respectively. For the other two subjects, we chose a Euclidean distance of ≤ 4 (sub_03) and ≤ 5 (sub_04) instead, corresponding to 11 or 7 observations, respectively. The observations labeled as most optimal are illustrated as orange dots in Fig. 3b.

*Criteria for the regressor modeling the least optimal stimuli combinations.* As the real-time stimuli selection using the Bayesian optimization method is aiming to maximize the expected improvement (see Methods), the least optimal stimuli combinations were sampled less frequently compared to the most optimal stimuli combinations. For this reason, we increased the Euclidean distance for all subjects. For three subjects (sub_01, sub_02 and sub_05), we selected a Euclidean distance of ≤ 4, corresponding to 5, 3 and 7 observations, respectively. For another subject (sub_03), we used a Euclidean distance ≤ 5, corresponding to 5 observations. As for sub_04 only one run was available, only a single observation was in close vicinity to the minimum. In this case, we additionally chose observations that were in close neighborhood to the coordinates of the second minimum of the predicted experiment parameter space, resulting in 3 observations entering the GLM.
