# Supplementary material for: The Automatic Neuroscientist: A framework for optimizing experimental design with closed-loop real-time fMRI
Source: Neuroimage. 2016 Apr 1;129:320–34. doi: 10.1016/j.neuroimage.2016.01.032 (PMC4819592; doi:10.1016/j.neuroimage.2016.01.032)
Supplement: Supplementary file 1 — Supplementary material [file mmc1.pdf]

Supplementary Material

The Automatic Neuroscientist: A framework for optimizing experimental design with closed-loop real-time fMRI

Romy Lorenz, Ricardo Pio Monti, Inês R. Violante, Christoforos Anagnostopoulos, Aldo A. Faisal, Giovanni Montana & Robert Leech

doi:10.1016/j.neuroimage.2016.01.032

Supplementary Table 1	Overview of visual parametric stimulus variation from no (1) to complex (10) visual input
Supplementary Table 2	Overview of auditory parametric stimulus variation from no (1) to complex (10) auditory input
Supplementary Figure 1	Five parametrically varied visual stimuli used in the study
Supplementary Figure 2	Results of offline analysis on data from Study 1 using Bayesian optimization
Supplementary Figure 3	Mean Euclidean distance of predicted optimum from hypothesized optimum for each subject when averaged across all available runs

Supplementary Tables

Supplementary Table 1. Overview of visual parametric stimulus variation from no (1) to complex (10) visual input

Visual complexity	Number of frames	Image size (pixel dimensions)	Image saturation (percentage)	2D Gaussian filter (SD)	Added Gaussian noise (variance)
1	-	-	same as baseline	same as baseline	same as baseline
2	1	65 x 116	1.7	8.9	8.9
3	3	125 x 221	2.8	7.8	7.8
4	6	184 x 327	4.6	6.7	6.7
5	11	243 x 432	7.7	5.6	5.6
6	21	303 x 538	12.8	4.5	4.5
7	39	362 x 644	21.2	3.4	3.4
8	72	422 x 749	35.3	2.3	2.3
9	133	481 x 855	58.7	1.2	1.2
10	250	540 x 960	original video	original video	original video

Supplementary Table 2. Overview of auditory parametric stimulus variation from no (1) to complex (10) auditory input

Auditory complexity	Number of bands for noise-vocoded speech	Added Gaussian noise (SD)
1	same as baseline	same as baseline
2	1	.08
3	2	.05
4	3	.03
5	4	.01
6	5	.008
7	6	.005
8	10	.003
9	20	.001
10	original sentence	original sentence

Supplementary Figures

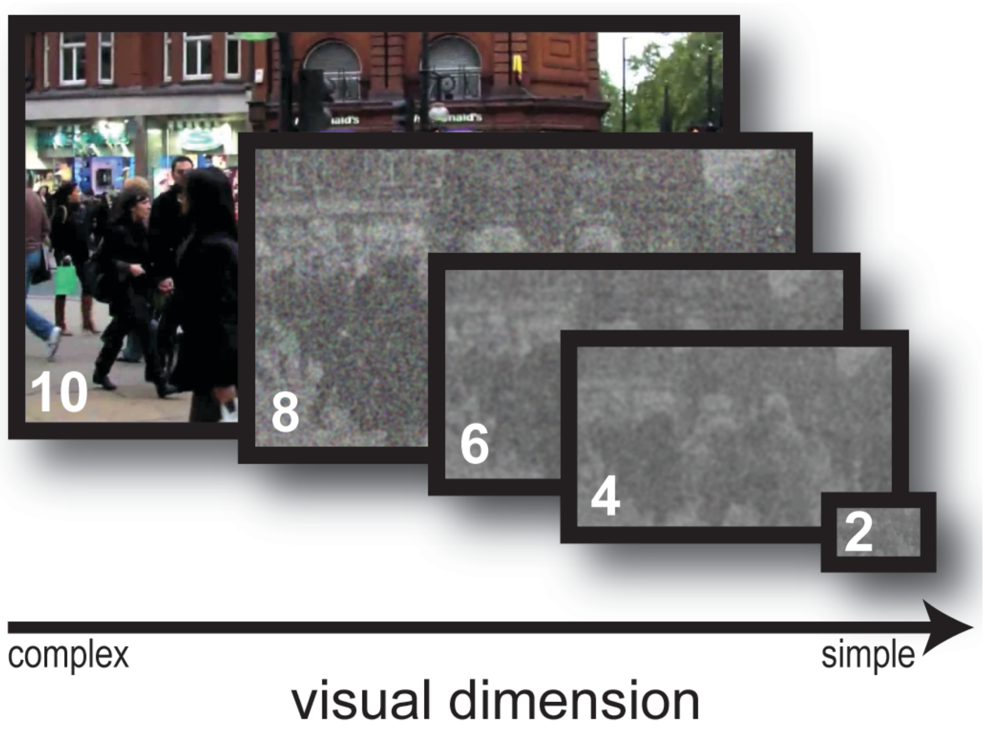

Supplementary Figure 1: Five parametrically varied visual stimuli used in the study. The visual stimuli are sorted according to their visual complexity from (10) complex (i.e., original video footage) to (2) very simple (i.e., highly modified). Note that (1) was same as baseline, i.e. a black background. Besides varying parameters such as image size, image saturation and spatial blurring as depicted here, we also varied the number of frames, in which a lower number of frames was subjectively experienced as a slower video.

.

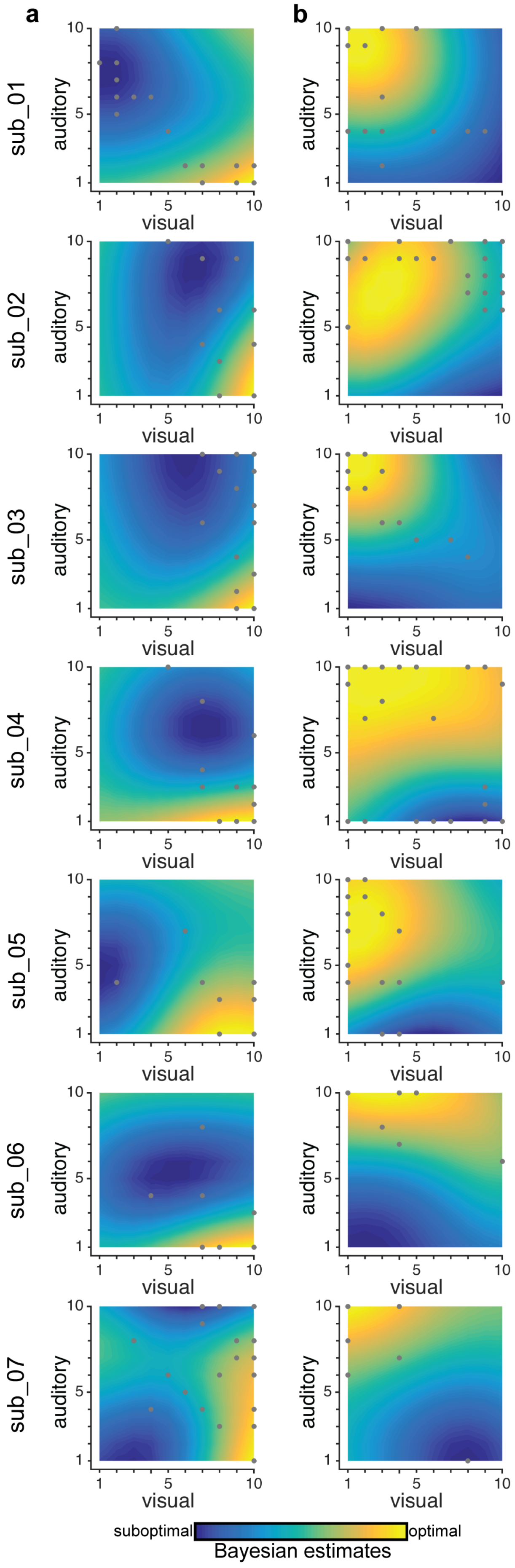

Supplementary Figure 2: Results of offline analysis on data from Study 1 using Bayesian optimization. The color bar represents the estimates by the Bayesian method on how optimal the experimental condition is for evoking the target brain state: the higher the predicted value, the more optimal the stimuli combination (yellow); the lower the predicted value, the less optimal the stimuli combination (dark blue). **(a)** Parameter space of each subject estimated on the run that optimized for target brain state (1), i.e. maximum lateral occipital with minimum superior temporal cortex activity. The hypothesized optimum is expected to be located in the bottom right corner (Fig. 2b). This was found by the Bayesian optimization in every subject as indicated by the maximum predicted values in this area of the grid (shown in yellow). **(b)** Parameter space of each subject estimated on the run that optimized for target brain state (2), i.e. minimum lateral occipital with maximum superior temporal cortex activity. The hypothesized optimum is expected to be located in the top left corner (Fig. 2b). Although not as confined mapped out as for target brain state (1), this was found in every subject. Parameter space estimates were based on all available observations in the respective run, illustrated as gray dots. Note that these results relied on the available observations that were proposed by the SPSA algorithm (collected in real-time); the superiority of the Bayesian optimization could only be validated in another real-time experiment.

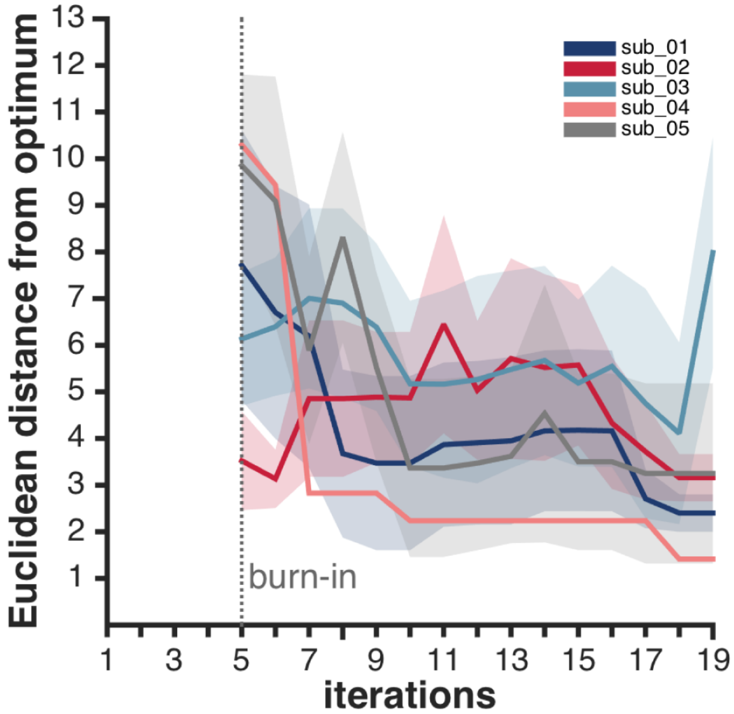

Supplementary Figure 3: Mean Euclidean distance of predicted optimum from hypothesized optimum for each subject when averaged across all available runs. As the first five iterations were used as a burn-in for a first estimate of the Bayesian model, they are not depicted here. Shaded areas represent the SEM. The data summarize results from all four runs for all individuals except for sub_04 who only completed a single run due to MRI technical failure during scanning. As can be seen, inter-run variability was high in all remaining four subjects.
