# Supplementary figures and images for: The Automatic Neuroscientist: A framework for optimizing experimental design with closed-loop real-time fMRI
Source: Neuroimage. 2016 Apr 1;129:320–34. doi: 10.1016/j.neuroimage.2016.01.032 (PMC4819592; doi:10.1016/j.neuroimage.2016.01.032)

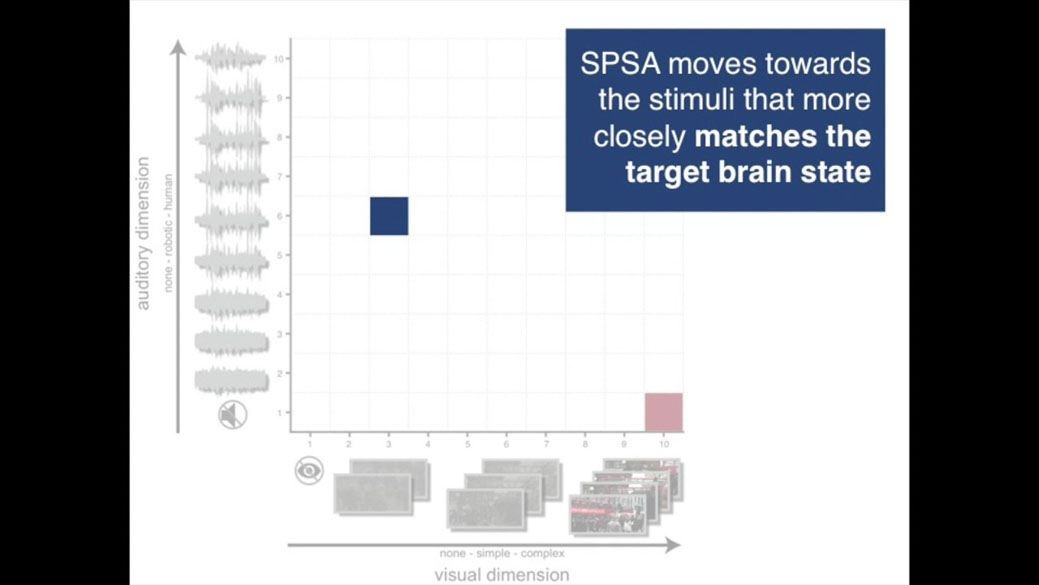

Supplement: Video 1 — The video shows how the stochastic approximation is performed using the SPSA algorithm. At each iteration, the SPSA randomly proposed two new audio–visual stimulus combinations within the parameter space where the objective is evaluated. These results are then used to obtain an approximation to the gradient, from which the algorithm proposes two new stimuli selections. This cycle continues until some stopping criterion is reached. In our work, convergence was based on an arbitrary threshold and defined as sampling the same combination of stimuli for three consecutive iterations (i.e., the same stimuli combination was chosen to be optimal on three consecutive iterations). If convergence occurred the scan was stopped. If convergence did not occur within 10 min, the experiment ended automatically in order to keep scanning time to a comfortable length for the participants. [file mmc2.jpg]

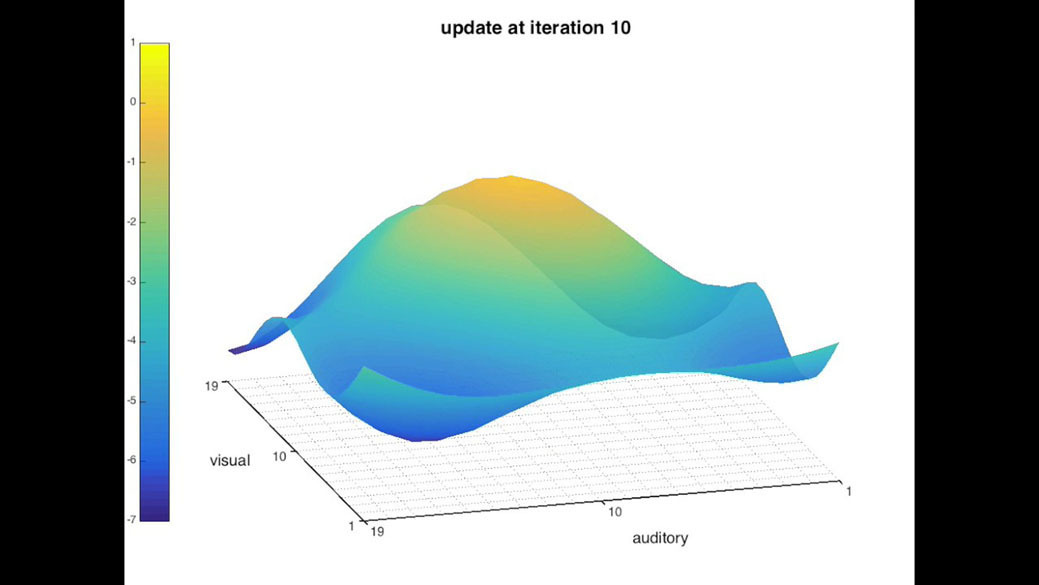

Supplement: Video 2 — The video shows how the Bayesian optimization algorithm is exploring the experiment parameter space over time and is learning the relationship between target brain state and audio–visual stimuli combinations over different iterations/observations. The height of the objective function represents the predictions by the Bayesian method on how optimal the experimental condition is for evoking the target brain state: the higher the predicted value, the more optimal the stimuli combination (yellow); the lower the predicted value, the less optimal the stimuli combination (dark blue). As can be seen, from iteration 12 on the algorithm seems to have obtained a global understanding of the experiment parameters space and keeps sampling the predicted optimum over multiple iterations, hence trying to maximize the expected improvement as described in the Methods section. Each run was automatically stopped after 19 observations. [file mmc3.jpg]
